# Associations between potassium, arterial stiffness, and risk of cardiovascular disease in the Jackson Heart Study: Potassium, arterial stiffness, and CVD risk

**DOI:** 10.1016/j.ajpc.2025.100955

**Published:** 2025-03-08

**Authors:** Ranee Chatterjee, Clemontina A Davenport, Ervin R. Fox, Ramachandran S. Vasan, Gary F Mitchell

**Affiliations:** a Department of Medicine, Duke University School of Medicine, Durham, NC, USA; b Department of Biostatistics and Data Science, Wake Forest University School of Medicine, Winston-Salem, NC, USA; c Department of Medicine, The University of Mississippi Medical Center, Jackson, MS, USA; d The University of Texas School of Public Health and the University of Texas Health Sciences Center, San Antonio, TX, USA; e Cardiovascular Engineering, Inc, Norwood, MA, USA

**Keywords:** Potassium, Arterial stiffness, Cardiovascular disease risk, Jackson heart study

## Abstract

**Background:**

Potassium (K) measures are associated with cardiovascular disease (CVD) risk factors, particularly blood pressure (BP). Arterial stiffness is a pre-clinical marker of CVD risk. We sought to study associations of K measures with arterial stiffness and CVD risk in a population at high-risk of CVD.

**Methods:**

We studied participants from the Jackson Heart Study (JHS), a longitudinal cohort of adults racially minoritized as Black, who were without CVD at Visit 1 (2000–2004). We compared characteristics between participants with low-normal (lowK) (≤4.0 mmol/L) vs. high-normal (highK) (>4.0 mmol/L) serum K. We used multivariable regression to examine associations of serum and dietary K at Visit 1 with arterial stiffness [brachial artery pulse pressure (PP) and carotid-femoral pulse wave velocity (CFPWV)], measured between 2012 and 2017, incident CVD overall over up to 15 years of follow-up, and individual CVD outcomes.

**Results:**

We included 4035 JHS participants in our analyses; mean age was 54 years, 64 % were female. Participants with highK as compared to lowK had lower mean baseline BP and had reduced arterial stiffness. In adjusted models, higher serum K (per standard deviation increase) was associated with lower CFPWV [estimate (95 % CI) -1.66 (-2.88, -0.44)]. There was a significant difference in cumulative incidence of CVD, with the highK group having lower risk (*P* = 0.047); however, we did not observe statistically significant associations between serum K and any CVD outcomes after multivariable adjustment. We found no significant associations between dietary K and arterial stiffness or incident CVD.

**Conclusions:**

In this cohort of Black adults, higher serum K was significantly associated with lower arterial stiffness. Further study is needed to assess the relationship between K's association with arterial stiffness and future CVD risk.

## Graphical abstract

**Figure:**
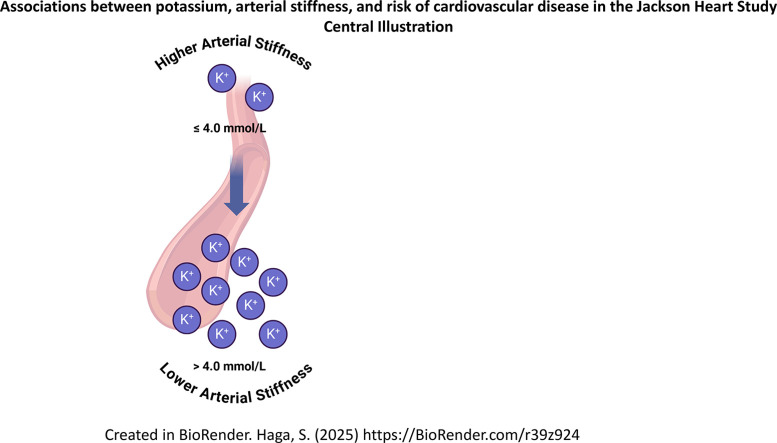

## 1 Introduction

Effective preventive measures are needed to reduce the risk of cardiovascular disease (CVD), particularly in Americans minoritized as Black, who are disproportionately affected by CVD and risk factors for CVD such as diabetes and hypertension (HTN) [1]. Potassium (K) deficiency is a potentially modifiable risk factor for CVD that is inexpensive to identify and treat and is broadly applicable. Potassium has been identified as a nutrient of concern due to its underconsumption and its association with health benefits [2,3] Studies have found significant associations between low K measures and increased risk of CVD, including heart disease and stroke as well as increased risk of diabetes, a strong CVD risk factor [[4], [5], [6], [7], [8], [9]]. Clinical trials have established that low K intake is directly linked to HTN [10]. Low K intake is highly prevalent, with <3 % of the US population achieving the adequate intake level recommended for dietary K from 2005, which was 4700mg/day [11,12] Moreover, studies indicate that Americans minoritized as Black have lower serum K measures and lower K intake than people of White race [13,14]

Accurate assessment of K status is difficult and requires whole body potassium counters, which is not feasible for studies. Serum K levels are tightly regulated and are impacted by factors such as renal function, endogenous hormonal regulation, and medications; and serum K levels are weakly correlated with dietary K intake, which is more closely correlated to urinary K measures. However, low-normal serum K levels have been found to reflect low total body stores of K and moderate K deficiency [12].

Prospective studies to measure the impact of moderate K deficiency and randomized clinical trials to assess the direct impact of treatments to correct K deficiency on CVD risk would require enrollment of large numbers of participants with follow-up over years to decades. However, a study centered on determining the impact of treatments to correct K deficiency on a marker of pre-clinical CVD is feasible for a smaller number of participants with a shorter follow-up period. Arterial stiffness is a well-studied marker that predicts the development of CVD [15,16] Measures of arterial stiffness include pulse wave velocity (PWV) and pulse pressure (PP), with PWV being considered the gold standard [17]. While PWV is strongly associated with age and blood pressure, it is also independently predictive of heart disease, stroke, and HTN, and is associated with other cardiometabolic risk factors, including glucose regulation [[15], [16], [17]].

We hypothesized that moderate K deficiency is associated with increased arterial stiffness and incident CVD in Black Americans. To study this hypothesis, we examined the associations of serum K and dietary K intake with arterial stiffness and incident CVD events in the Jackson Heart Study (JHS) cohort.

## 2 Methods

### 2.1 Study participants

The JHS is a community-based prospective cohort study of 5306 Black adults ranging in age from 21 to 84 years at the time of recruitment from the tri-county area (Hinds County, Rankin County and Madison County) that includes the Jackson, Mississippi metropolitan area. This cohort was designed to study the prevalence and incidence of CVD risk factors in a sample of Black Americans. Participants were recruited beginning in 2000, with Visit 1 data collected between 2000 and 2004; eligibility criteria have been described previously [18]. Participants came for in-person visits approximately every 4 years through 2012. At study visits, participants underwent self-administered and interview-administered questionnaires, anthropometric measurements, and measurement of vital signs, as well as radiologic and laboratory evaluations. A vascular function ancillary study was conducted in a sub-cohort of participants between 2012 and 2017, with additional measures described below. Annual phone calls to participants were conducted to assess for health events, including outcomes of CVD. Reported outcomes were then verified through review of medical records [19]. For the present analyses, those with any history of myocardial infarction, stroke, coronary heart disease, or heart failure at Visit 1 were excluded. Further exclusions for missing data were model-dependent so that all analyses were performed on a subset of our defined analytic cohort, rather than a separate sample of participants. Institutional review boards at each of the participating institutions approved the study.

### 2.2 Main exposures

Our primary exposures for these analyses were serum K and dietary K intake. Serum K was measured in all participants at Visit 1 only. For serum and blood measures, participants underwent venipuncture in a fasting state. Blood samples were centrifuged, portioned into aliquots, frozen, and stored in a −70 °Celsius freezer in a central laboratory. Serum K was measured with a direct electrode potentiometric assay (Vitros 950 or 250 analyzer; Ortho-Clinical Diagnostics) [20]. Dietary intake of nutrients, including K and total energy intake, was assessed in all participants at the Visit 1 exam. Participants completed a 158-item food-frequency questionnaire, which was tailored and validated to be culturally appropriate for this population and was administered face-to-face by trained interviewers minoritized as Black [21,22] Dietary intake was also categorized as adequate (≥3600 mg/day for males and ≥2600 mg/day for females) or inadequate (<3600 mg/day for males and <2600 mg/day for females).

### 2.3 Outcomes

Our primary outcomes were peripheral PP from Visit 1; tonometry-based PP as part of the vascular function ancillary study (2012–2017); carotid-femoral pulse wave velocity (CFPWV) from the same ancillary study (2012–2017); and adjudicated incident CVD, defined as the occurrence of any of the following events during follow-up, which was truncated at a maximum of 15 years. Myocardial infarction, stroke, and coronary heart disease events were adjudicated through December 31, 2016. Heart failure events were available from 2005 to December 31, 2016 [19,23]

Peripheral PP was calculated from brachial artery blood pressure measurements taken at Visit 1. Blood pressure was measured in a seated position with the use of calibrated equipment and a standardized protocol; and was recorded as the mean of two blood pressure readings taken 30 s apart and after an initial 5 min of rest [[24], [25], [26]]. The average of the two blood pressure readings was used; and peripheral PP was calculated as systolic blood pressure minus diastolic blood pressure. Tonometry PP was assessed from the supine blood pressure taken at the time of arterial tonometry. Calibrated blood pressure data were used for these measures as described previously [27].

For measurements of CFPWV, participants were placed in a supine position for 10 min of rest. Arterial tonometry and ECG were obtained from the brachial, radial, femoral, and carotid arteries with a custom transducer. Body surface measurements were assessed from the suprasternal notch to brachial, radial, femoral, and carotid recording sites. All data were digitized during the primary acquisition (ECG and tonometry pressures at 1000 Hz, audio at 12 kHz, and video at 30 frames/s), transferred to CD-ROM, and shipped to the Core Laboratory at Cardiovascular Engineering, Inc. for analysis. CFPWV was calculated similarly to prior studies by dividing the carotid-femoral transit distance (the difference in body surface measurements from the suprasternal notch to the femoral and carotid sites) by the carotid-femoral transit time (measured using the foot of the carotid and femoral waveforms) [28,29]

### 2.4 Covariates

Covariates were obtained at Visit 1. Many were obtained through self-report, including age, sex, annual household income, education level, physical activity, and smoking history. Anthropometric measurements and vital signs (body mass index, estimated glomerular filtration rate, heart rate, and blood pressure) were obtained using a standardized protocol, which has been previously described [26]. The use of medications was assessed at in-person study visits; participants were asked to bring in all medications taken during the prior two weeks and research staff reviewed and recorded them. Diabetes status was determined by a combination of self-report of history of diabetes, laboratory values (fasting glucose ≥126 mg/dL or HbA1c levels ≥6.5 %), and use of diabetes medications. Physical activity was measured with the use of a validated 30-item interviewer-administered survey [30].

### 2.5 Statistical analysis

We categorized Visit 1 serum K measures into low-normal (lowK) (≤4.0 mmol/L) and high-normal (highK) (>4.0 mmol/L), given that physiologic studies have demonstrated that low-normal serum K levels could reflect low total body stores of potassium [12]. We examined the baseline characteristics of the included participants by serum K categories, including tonometry-based measurements such as CFPWV. Serum K was modeled both as a binary variable and as a continuous variable in our models, which are described further below.

To determine the association between serum K and both peripheral and tonometry-based PPs, we fit minimally (model 1) and more fully-adjusted (model 2) multivariable linear regression models, adjusting for potential confounding at Visit 1. Model 1 included age, age^2^, sex, age-sex interaction, age^2^-sex interaction, and heart rate, while model 2 included age, age^2^, sex, age-sex interaction, age^2^-sex interaction, heart rate, body mass index (BMI), smoking status, use of statin medication, use of antihypertensive medication, diabetes diagnosis/medication use, and use of hormone replacement therapy (HRT) in women. The age^2^ terms were added because previous studies have shown that pulse pressure was the only measure of aortic stiffness to have a strong non-linear association with age [31]. Tonometry PP models were adjusted for tonometry-based variables measured when available (e.g., heart rate).

In analyses to determine the association between serum K and CFPWV, we used a negative inverse transformation for CFPWV (niCFPWV) to optimally address the skewed distribution of raw CFPWV measures, such that a higher value was indicative of more arterial stiffness. Model 1 included age and sex, and tonometry mean arterial pressure and heart rate at the ancillary study visit (2012–2017). Model 2 included age, sex, age-sex interaction, tonometry mean arterial pressure, tonometry heart rate, BMI, smoking status, use of statin medication, use of antihypertensive medication, diabetes diagnosis/medication use, and use of HRT in women.

We estimated the cumulative incidence of CVD events in the presence of death as a competing risk and compared the incidence between those in the highK vs lowK groups using a chi-squared test. We used minimally and more fully adjusted sub-distribution hazards models to explore the relationship between serum K and incident CVD. Model 1 included age, sex, mean arterial pressure, and heart rate. Model 2 additionally included age-sex interaction, BMI, smoking status, use of statins, use of antihypertensive medications, diabetes diagnosis/medication use, and use of HRT in women.

Similar models as above were used to assess the associations between dietary K intake and each of the four outcomes, with additional adjustment for total energy intake. Dietary K was modeled both continuously and dichotomized into adequate vs. inadequate intake.

### 2.6 *Post hoc* analyses

After reviewing the results of our primary analyses, we repeated the analyses with serum K categorized into quintiles in order to explore more granular differences between serum K groups on the outcomes of interest. We assessed the interaction effect of serum K (both continuous and low-normal vs normal) and diuretic use on our outcomes of interest. We also repeated the sub-distribution hazard models to assess the association between K and time to each CVD event type separately (i.e., myocardial infarction, stroke, or heart failure).

## 3 Results

Of the 5306 JHS participants, we excluded 1211 with a history of any CVD or were missing CVD information and 60 with missing serum K at Visit 1. Characteristics of our middle-aged, predominantly female sample are presented in Table 1. A CONSORT diagram is presented as **Supplementary Figure 1**. Participants with highK compared to lowK had lower mean BMI. They were less likely to be taking statins, HRT, and antihypertensive medications; and less likely to have diabetes. Additionally, participants in the highK group compared to the lowK group had lower blood pressure, lower seated (clinic) and supine (tonometry visit) PP, and lower CFPWV on average. They also differed in socioeconomic characteristics (Table 1). The median time difference between measures of K and measures of CFPWV was 8 years.

**Table 1:** Demographic and clinical characteristics and measures of Jackson Heart Study (JHS) participants stratified by serum potassium (K) group.

	Overall (*N* = 4035)	LowK (*K* ≤ 4.0 mmol/L) (*N* = 1080)	HighK (*K* > 4.0 mmol/L) (*N* = 2955)
**Visit 1 Variables and Measures**#			
**Age (years)**	53.9 (12.5)	55.7 (11.9)	53.3 (12.7)
**Female sex**	2589 (64.2)	797 (73.8)	1792 (60.6)
Income*			
Poor	456 (11.3)	127 (11.8)	329 (11.1)
Lower-middle	792 (19.6)	232 (21.5)	560 (19.0)
Upper-middle	1057 (26.2)	298 (27.6)	759 (25.7)
Affluent	1130 (28.0)	253 (23.4)	877 (29.7)
**Education**†			
Less than high school	633 (15.7)	198 (18.3)	435 (14.7)
HS graduate / GED	796 (19.7)	214 (19.8)	582 (19.7)
Some college or more	2593 (64.3)	662 (61.3)	1931 (65.3)
**BMI (kg/m^2^)**	31.7 (7.1)	32.5 (7.2)	31.3 (7.1)
**Smoking status**			
Never	2824 (70.0)	765 (70.8)	2059 (69.7)
Former	735 (18.2)	223 (20.6)	512 (17.3)
Current	467 (11.6)	90 (8.3)	377 (12.8)
**Physical activity**‡			
Poor	1894 (46.9)	543 (50.3)	1351 (45.7)
Intermediate	1322 (32.8)	327 (30.3)	995 (33.7)
Ideal	815 (20.2)	208 (19.3)	607 (20.5)
**Total dietary energy (kcal)**	8660 (3710)	8310 (3500)	8790 (3780)
**Statins**	446 (11.1)	140 (13.0)	306 (10.4)
**Hormone replacement therapy**	704 (17.4)	249 (23.1)	455 (15.4)
**Antihypertensive medications**	1925 (47.7)	727 (67.3)	1198 (40.5)
**Diabetes medications**	519 (12.9)	146 (13.5)	373 (12.6)
**Diabetes status**§			
No diabetes	1847 (45.8)	448 (41.5)	1399 (47.3)
Prediabetes	1369 (33.9)	384 (35.6)	985 (33.3)
Diabetes	817 (20.2)	248 (23.0)	569 (19.3)
**eGFR (ml/min/1.73m2)**	86.7 (17.4)	87.2 (17.0)	86.6 (17.5)
**CKD status by eGFR**			
G1 or G2 (eGFR ≥ 60)	3784 (93.8)	1012 (93.7)	2772 (93.8)
G3 (30 ≤ eGFR < 60)	232 (5.7)	66 (6.1)	166 (5.6)
G4 (15 ≤ eGFR < 30)	13 (0.3)	1 (0.1)	12 (0.4)
G5 (eGFR < 15)	5 (0.1)	1 (0.1)	4 (0.1)
**Serum K (mmol/L)**	4.28 (0.394)	3.80 (0.208)	4.45 (0.287)
**Dietary K intake (mg)**	2540 (1090)	2480 (1090)	2560 (1090)
**Adequate dietary K intake**^**€**^	1078 (26.7)	291 (26.9)	787 (26.6)
**ECG heart rate (bpm)**	64.1 (10.3)	64.4 (10.6)	64.0 (10.2)
**Systolic BP (mmHg)**	127 (16.2)	129 (16.7)	126 (16.0)
**Diastolic BP (mmHg)**	76 (8.5)	76 (8.7)	76 (8.4)
**Mean arterial pressure (mmHg)**	93 (9.6)	94 (9.8)	92 (9.5)
**Peripheral pulse pressure (mmHg)**	51 (14.0)	53 (14.5)	50 (13.8)

**Ancillary Sub-study Measures (2012–2017)**#			
**Tonometry heart rate**	65.7 (10.3)	65.6 (9.9)	65.7 (10.4)
**Tonometry systolic BP**	136 (18.1)	138 (19.1)	136 (17.7)
**Tonometry diastolic BP**	73 (10.5)	73 (11.1)	73 (10.3)
**Tonometry mean arterial pressure**	99 (12.0)	100 (13.0)	99 (11.6)
**Tonometry peripheral pulse pressure**	63 (16.5)	65 (16.5)	63 (16.5)
**CFPWV (m/s)**||			
Mean (SD)	11.1 (4.35)	11.8 (4.67)	10.8 (4.20)
Median [Q1, Q3]	10.0 [8.20, 12.6]	10.6 [8.75, 13.7]	9.80 [8.04, 12.2]
(Min, Max)	(3.23, 30.0)	(3.35, 30.0)	(3.23, 30.0)
**Negative inverse CFPWV** **(−1000/(m/s))**			
Mean (SD)	−101 (32.5)	−95.6 (32.1)	−103 (32.3)
Median [Q1, Q3]	−100 [−122, −79.1]	−94.7 [−114, −72.9]	−102 [−124, −82.0]
(Min, Max)	(−310, −33.3)	(−299, −33.3)	(−310, −33.3)

# Values are given as mean (SD) for continuous variables and n (%) for categorical variables.

⁎ Income defined as the following: Poor (<1 times the poverty level), Lower-middle (1 to 1.5 times the poverty level), Upper-middle (>1.5 to <3.5 times the poverty level), Affluent (≥3.5 times the poverty level);.

† Education of “some college or more” defined as attended vocational school, trade school, or college;.

‡ Physical activity defined as the following: "Poor: 0 mins of moderate physical activity; and 0 mins of vigorous physical activity; Intermediate: 0 < mins of moderate physical activity < 150; or 0 < mins of vigorous physical activity < 75; or 0 < mins of combined moderate and vigorous physical activity < 150; Ideal: mins of moderate physical activity ≥ 150; or mins of vigorous physical activity ≥ 75; or mins of combined moderate and vigorous physical activity ≥ 150;.

§ No diabetes defined as: HbA1c < 5.7 %; and fasting plasma glucose < 100 mg/dL; and no report of taking diabetes medications; Pre-diabetic or at risk of diabetes defined as: 5.7 % ≤ HbA1c < 6.5 %; or 100 mg/dL ≤ Fasting Glucose < 126 mg/dL; and no report of taking diabetes medications; Diabetic defined as: fasting glucose ≥ 126 mg/dL; or HbA1c ≥ 6.5 %; or report of taking diabetes medications.

|| CFPWV= carotid femoral pulse wave velocity^€^Adequate K intake defined as >3600 mg/day for men and >2600 mg/day Column percentages may not sum to 100 due to missing values.

In model 1 and model 2, serum K was significantly and inversely associated with peripheral PP (Table 2). We also found that those with highK had significantly lower niCFPWV on average compared to those with lowK in model 1. Additionally, in both models 1 and 2, each SD increase in serum K was significantly associated with lower transformed CFPWV (Table 2). We found no significant associations between serum K and tonometry-based PP.

**Table 2:** Associations between serum and dietary potassium (K) and measures of arterial stiffness and risk of cardiovascular disease (CVD).

	Visit 1 Peripheral PP* Estimate (95 % CI)	Tonometry Peripheral PP* Estimate (95 % CI)	Negative-inverse CFPWV† Estimate (95 % CI)	Incident CVD HR (95 % CI)
**Serum K**	*N* = 3972	*N* = 2368	*N* = 1833	*N* = 3972
HighK vs LowK (Model 1)	**−1.5 (−2.4, −0.7)**	−0.9 (−2.3, 0.5)	**−3.3 (−6.0, −0.6)**	0.9 (0.7, 1.1)
HighK vs LowK (Model 2)	**−1.1 (−2.0, −0.2)**	−0.1 (−1.5, 1.3)	−2.2 (−5.0, 0.5)	1.0 (0.8, 1.2)
Per SD (Model 1)	**−0.6 (−1.0, −0.2)**	−0.3 (−1.0, 0.3)	**−2.1 (−3.3, −0.9)**	1.0 (0.9, 1.1)
Per SD (Model 2)	−0.3 (−0.8, 0.1)	0.1 (−0.6, 0.8)	**−1.7 (−2.9, −0.4)**	1.0 (0.9, 1.1)
**Dietary K intake**	*N* = 3636	–	*N* = 1697	*N* = 3636
Per 100 mg (Model 1)	−0.0 (−0.1, 0.0)	–	−0.1 (−0.3, 0.1)	1 (1.0, 1.0)
Per 100 mg (Model 2)	−0.0 (−0.1, 0.0)	–	−0.1 (−0.3, 0.1)	1.0 (1.0, 1.0)
Adequate vs. inadequate intake (model 1)	−0.3 (−1.4, 0.9)	–	−1.2 (−4.4, 2.0)	1.2 (0.9, 1.5)
Adequate vs. inadequate intake (model 2)	−0.5 (−1.7, 0.6)	–	−1.4 (−4.6, 1.9)	1.1 (0.8, 1.5)

LowK defined as serum *K* ≤ 4.0 mmol/L.

HighK defined as serum *K* > 4.0 mmol/L.

Estimates in bold are statistically significant at an alpha level of 0.05.

⁎ PP= pulse pressure.

† CFPWV= carotid femoral pulse wave velocity – Models were not fit *a priori* Models 1: For PP- adjusted for age, age^2^, sex, age-sex interaction, age^2^-sex interaction, and heart rate; for CFPWV- adjusted for age and sex and tonometry mean arterial pressure and heart rate; for CVD- age, sex, mean arterial pressure, and heart rate; also total dietary energy intake (kcal) for dietary K intake models Models 2: For PP- adjusted for age, age^2^, sex, age-sex interaction, age^2^-sex interaction, and heart rate, body mass index, active smoking, statin medication, antihypertensive medication, diabetes diagnosis/medication use, and hormone replacement therapy (HRT) in women; for CFPWV- age, sex, age-sex interaction, tonometry mean arterial pressure, tonometry heart rate, body mass index, active smoking, statin medication, antihypertensive medication, diabetes diagnosis/medication use, and HRT in women; For CVD- age, sex, mean arterial pressure, and heart rate, age-sex interaction, BMI, smoking status, use of statins, use of antihypertensive medications, diabetes status, and use of HRT in women; also total dietary energy intake (kcal) for dietary K intake models.

**Fig. 1:**
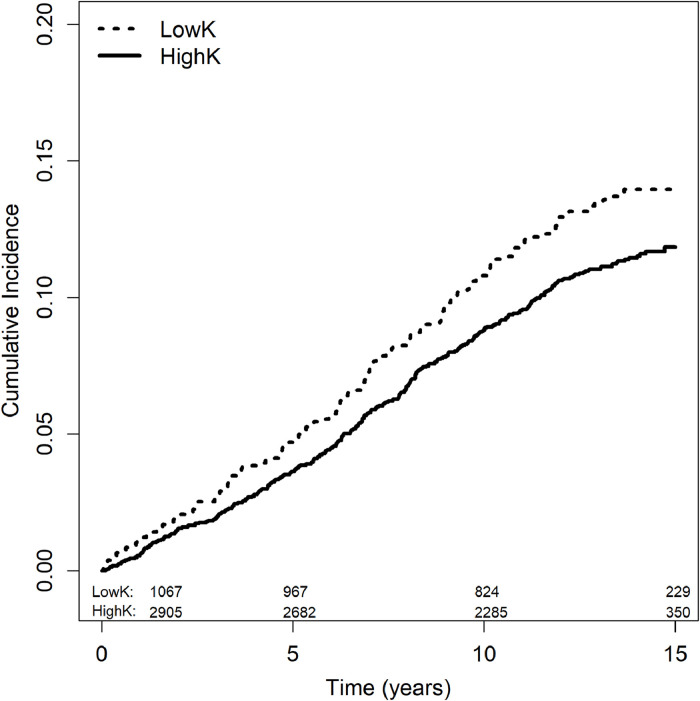
Estimated Cumulative Incidence of Cardiovascular Disease Events by Serum Potassium (K) Group Chi-squared test for significance, *P* = 0.047Number of participants analyzed at each time point by lowK and highK group are shown.

There were 473 CVD events over 15 years, with 329 (11.1 %) events occurring in the highK group and 144 (13.3 %) events occurring in the lowK group. Those with lowK had higher unadjusted cumulative incidence of CVD compared to those with highK (*P* = 0.047) (Fig. 1). However, we found no significant associations between serum K measures and incident CVD after multivariable adjustment. We also found no significant associations between dietary K and any measures of arterial stiffness nor incident CVD.

In *post hoc* analyses with serum K modeled in quintiles, we found that compared to those with a serum *K* ≤ 4.0 mmol/L, those with K between >4.2 and ≤4.4 mmol/L and those with K between >4.4 and ≤4.6 had significantly lower peripheral PP in models 1 and 2. Those with K between >4.4 and ≤4.6 mmol/L and those with K between >4.6 and ≤6.2 had significantly reduced CFPWV in model 1, and those with serum K between >4.6 and ≤6.2 mmol/L also had significantly reduced CFPWV in model 2 (Table 3).

**Table 3:** Associations between quintiles of serum potassium (K) and measures of arterial stiffness and risk of cardiovascular disease (CVD).

	**Visit 1** Peripheral PP* Estimate (95 % CI)	**Tonometry** Peripheral PP* Estimate (95 % CI)	**Negative-inverse** **CFPWV**† **Estimate (95 % CI)**	**Incident CVD** **HR (95 % CI)**‡
**Model 1**				
2.5 ≤ *K* ≤ 4	(ref)	(ref)	(ref)	(ref)
4 < *K* ≤ 4.2	−0.9 (−2.0, 0.3)	−0.5 (−2.2, 1.3)	−2.1 (−5.4, 1.3)	1.1 (0.8, 1.4)
4.2 < *K* ≤ 4.4	**−1.9 (−3.0, −0.8)**	−1.2 (−2.9, 0.5)	−2.3 (−5.7, 1.1)	0.9 (0.7, 1.2)
4.4 < *K* ≤ 4.6	**−2.3 (−3.5, −1.1)**	−0.6 (−2.5, 1.3)	**−4.3 (−7.9, −0.7)**	0.8 (0.6, 1.1)
4.6 < *K* ≤ 6.2	**−1.3 (−2.6, −0.0)**	−1.4 (−3.3, 0.6)	**−5.3 (−9, −1.5)**	0.9 (0.6, 1.2)
**Model 2**				
2.5 ≤ *K* ≤ 4	(ref)	(ref)	(ref)	(ref)
4 < *K* ≤ 4.2	−0.7 (−1.8, 0.5)	−0.0 (−1.8, 1.8)	−1.4 (−4.7, 2.0)	1.1 (0.8, 1.4)
4.2 < *K* ≤ 4.4	**−1.4 (−2.5, −0.3)**	−0.4 (−2.1, 1.4)	−1.1 (−4.6, 2.4)	1.0 (0.8, 1.3)
4.4 < *K* ≤ 4.6	**−1.6 (−2.8, −0.4)**	0.5 (−1.4, 2.4)	−3.1 (−6.7, 0.6)	0.9 (0.7, 1.2)
4.6 < *K* ≤ 6.2	−0.8 (−2.1, 0.5)	−0.5 (−2.5, 1.6)	**−4.4 (−8.2, −0.6)**	1.0 (0.7, 1.3)

*K* = serum K in mmol/L.

Estimates in bold are statistically significant at an alpha level of 0.05.

⁎ PP= pulse pressure.

† CFPWV= carotid femoral pulse wave velocity.

‡ HR= hazard ratio Models 1: For PP- adjusted for age, age^2^, sex, age-sex interaction, age^2^-sex interaction, and heart rate; for CFPWV- adjusted for age and sex and tonometry mean arterial pressure and heart rate; for CVD- age, sex, mean arterial pressure, and heart rate Models 2: For PP- adjusted for age, age^2^, sex, age-sex interaction, age^2^-sex interaction, and heart rate, body mass index, active smoking, statin medication, antihypertensive medication, diabetes diagnosis/medication use, and hormone replacement therapy (HRT) in women; for CFPWV- age, sex, age-sex interaction, tonometry mean arterial pressure, tonometry heart rate, body mass index, active smoking, statin medication, antihypertensive medication, diabetes diagnosis/medication use, and HRT in women; For CVD- age, sex, mean arterial pressure, and heart rate, age-sex interaction, BMI, smoking status, use of statins, use of antihypertensive medications, diabetes status, and use of HRT in women.

We found a significant interaction effect between serum K and the use of diuretics on peripheral PP when serum K was modeled continuously and categorically (both *P* = 0.001). Those in the highK group had significantly lower peripheral PP than those in the lowK group among those with no diuretic use. In models for tonometry PP and niCFPWV, there were no statistically significant interactions between serum K and diuretic use (Table 4). We observed a potential interaction effect of diuretic use on the association between serum K group and incident CVD (*P* = 0.046). Those in the highK group compared to lowK tended to have a lower hazard of incident CVD among those with no diuretic use (HR [95 % CI]: 0.83 [0.61, 1.11]), but a higher hazard among those with diuretic use (1.26 [0.94, 1.68]), neither of which was statistically significant (Table 4). There were no significant associations found between serum K, either as a continuous or categorical variable, or dietary K with any of the individual CVD outcomes of CHD, stroke, or heart failure (Table 5).

**Table 4:** Associations^‡^ between serum potassium (K) and measures of arterial stiffness and risk of cardiovascular disease (CVD) stratified by use of diuretics.

	**No Diuretic Use** **(*N*=2829)**	**Diuretic Use** **(*N*=1206)**	
	**HighK vs LowK**	**HighK vs LowK**	***P*-interaction**
Visit 1 Peripheral PP*	**−2.6 (−3.8, −1.4)**	0.4 (−1.0, 1.9)	**0.001**
**Tonometry Peripheral PP**	−1.1 (−2.9, 0.7)	1.0 (−1.3, 3.3)	0.140
**Negative-inverse CFPWV**†	**−4 (−7.5, −0.5)**	−0.7 (−5.0, 3.7)	0.233
**Incident CVD**	0.8 (0.6, 1.1)	1.3 (0.9, 1.7)	**0.046**
	**Per SD**	**Per SD**	***P*-interaction**
**Visit 1 Peripheral PP**	**−1.0 (−1.5, −0.4)**	0.4 (−0.3, 1.1)	**0.001**
**Tonometry Peripheral PP**	−0.3 (−1.1, 0.5)	0.6 (−0.6, 1.9)	0.177
**Negative-inverse CFPWV**	**−2.4 (−3.8, −0.9)**	−0.9 (−3.2, 1.4)	0.248
**Incident CVD [HR (95 % CI)]**‡	1.0 (0.8, 1.1)	1.1 (1.0, 1.3)	0.120

Estimates in bold are statistically significant at an alpha level of 0.05.

LowK defined as serum *K* ≤ 4.0 mmol/L.

HighK defined as serum *K* > 4.0 mmol/L.

⁎ PP= pulse pressure.

† CFPWV= carotid femoral pulse wave velocity.

‡ HR= hazard ratio Models adjusted for: For PP- adjusted for age, age^2^, sex, age-sex interaction, age^2^-sex interaction, and heart rate, body mass index, active smoking, statin medication, antihypertensive medication, diabetes diagnosis/medication use, and hormone replacement therapy (HRT) in women; for CFPWV- age, sex, age-sex interaction, tonometry mean arterial pressure, tonometry heart rate, body mass index, active smoking, statin medication, antihypertensive medication, diabetes diagnosis/medication use, and HRT in women; For CVD- age, sex, mean arterial pressure, and heart rate, age-sex interaction, BMI, smoking status, use of statins, use of antihypertensive medications, diabetes status, and use of HRT in women.

**Table 5:** Associations between serum potassium (K) and dietary K with incident cardiovascular disease outcomes.

	**Coronary Heart Disease**	**Heart Failure**	**Stroke**
Serum K			
**number of events/N**	143/3972	287/3972	143/3972
**HighK vs LowK** **HR (95 % CI)**	1.0 (0.7, 1.4)	0.9 (0.7, 1.2)	1.2 (0.8, 1.8)
**Serum K per SD increase**	1.2 (1.0, 1.4)	1.0 (0.9, 1.2)	1.0 (0.9, 1.2)
**Dietary K**			
**number of events/N**	132/3636	260/3636	136/3636
**Dietary K per SD increase**	1.0 (1.0, 1.0)	1.0 (1.0, 1.0)	1.0 (1.0, 1.0)

LowK defined as serum *K* ≤ 4.0 mmol/L.

HighK defined as serum *K* > 4.0 mmol/L.

Models adjusted for: age, sex, mean arterial pressure, and heart rate, age-sex interaction, BMI, smoking status, use of statins, use of antihypertensive medications, diabetes status, and use of HRT in women.

## 4 Discussion

In our analyses, we found that JHS participants with higher serum K levels had significantly lower arterial stiffness as assessed by both peripheral PP and CFPWV; in unadjusted analyses, higher serum K was also associated with lower incidence of CVD. We did not find significant associations between serum K and incident CVD after multivariable adjustments; and we found no significant associations between dietary K intake and arterial stiffness or incident CVD. In exploratory *post hoc* analyses, we found evidence of an interaction effect of diuretic use on the association between serum K group and arterial stiffness measured by peripheral PP and the association between serum K group and incident CVD.

The association between serum K and arterial stiffness has biological plausibility. K concentrations directly impact arterial stiffness based on findings from basic science studies. Low K concentrations inhibited arterial smooth muscle and endothelium-dependent vasodilation, stimulated hypertrophy of vascular smooth muscle cells, and increased free-radical formation by vascular cells. Greater K concentrations, within a physiologic range, reversed all of the foregoing effects [[32], [33], [34]]. In human studies, the effects of K supplementation on arterial stiffness have been assessed in only a few previous trials. In a meta-analysis of seven clinical trials, K supplementation was associated with improvements in PP, but not in the other measures, including PWV and augmentation index [35]. However, none of these trials were conducted in the US among participants racialized as Black or among those of African descent. A cross-sectional cohort study from South Africa of participants of African descent found that higher K intake, as reflected by a lower urinary sodium/potassium ratio, was associated with lower PP, independent of blood pressure [36]. A US study of the Dietary Approaches to Stop Hypertension (DASH) diet, of which one component is the 2005 adequate intake level of K intake, found significant improvement in PWV [37].

In addition to direct effects on the arterial wall, treatment of K deficiency with K supplementation may improve arterial wall stiffness by mitigating CVD risk factors that impact arterial stiffness, including blood pressure and glucose metabolism. Meta-analyses of large clinical trials found significant blood pressure-lowering effects with K supplementation; greater blood pressure-lowering occurred in Black participants, among people with higher sodium intake, and with higher baseline BP [38]. A meta-analysis of cohort studies found low serum K to be a significant risk factor for diabetes [9]. A prior analysis of the JHS cohort found that lower serum K levels were associated with increased diabetes risk and that this association was independent of aldosterone levels among those participants with aldosterone levels within the normal range, which included the majority of the cohort [8]. Small trials of healthy participants demonstrated that low K can impair insulin secretion [[39], [40], [41]]. In a pilot RCT of Black adults with prediabetes, potassium chloride (KCl) lowered fasting glucose and branched-chain amino acids (BCAAs) levels, which are metabolomic biomarkers associated with insulin resistance and risk of CVD [42,43] The precise mechanisms through which K supplementation may improve blood pressure and glucose metabolism, however, have not been elucidated.

Given the high prevalence of low K intake and the high prevalence of CVD and CVD risk factors, K could be a modifiable risk factor for CVD with widespread impact. A combined cohort study found that higher dietary K intake, as reflected by increased urinary K, was associated with a significantly lower risk of CVD over a median of 9 years of follow-up; those in the highest quartile compared to the lowest had a 31 % lower risk of CVD [44]. The Salt Substitute and Stroke Study (SSaSS) trial, an intervention trial for primary and secondary prevention conducted in rural China, compared the effects of a salt substitute containing 25 % potassium chloride (KCl) compared to regular salt (100 % sodium chloride) on risk of stroke among Chinese adults who had a history of stroke or who were age 60 and older and had hypertension [45]. This study found significant reductions in CVD risk among those who received the KCl salt substitute, with a 14 % lower risk of stroke, 13 % lower risk of major CVD events, as well as a 12 % lower risk of death, over 5 years of follow-up. In sub-group analyses, there was a significantly reduced risk of stroke among those in the SSaSS trial without a prior history of stroke [45], suggesting that there are benefits to potassium supplementation for primary prevention of CVD. Based in large part on the results of the SSaSS trial, the American Heart Association suggests use of KCl salt substitutes as a 2a recommendation on diet quality for the primary prevention of stroke [46].

Arterial stiffness is an important intermediate outcome that is strongly associated with CVD risk [15]. As reflected by atherosclerotic lesions and medical history, in cross-sectional studies, increased PWV was associated with a greater prevalence of CVD, and these associations were consistent in Black, White, and Asian individuals [17].^,^ [47] In longitudinal cohorts, aortic stiffness predicted CVD events, including events related to heart disease, stroke, and CVD-related mortality. Importantly, this association was independent of traditional CVD risk factors, including blood pressure, glucose measures, and lipids [[15], [16], [17]]. In a meta-analysis of 19 studies, including US and European studies, a standard deviation higher value for CFPWV was associated with an increased risk of CVD events (relative risk [95 % CI]: 1.25 [1.19, 1.31]) and CVD-related mortality (relative risk [95 % CI]: 1.23 [1.15, 1.31]) [48]. In an earlier meta-analysis, every 1 m/s increase in aortic PWV was associated with 14 % increase in risk of CV events after adjustment for age, sex, and other risk factors [15]. Clinical trials have tested interventions, including lifestyle interventions and pharmacologic interventions, on measures of arterial stiffness. In a long-term clinical trial of participants with end-stage renal disease, interventions resulting in favorable PWV changes predicted improvements in mortality [49].

Arterial stiffness could potentially be a good intermediate outcome when developing interventions to reduce racial disparities in CVD risk. In a meta-analysis of studies measuring arterial stiffness in healthy Black and White adults, Black adults had greater arterial stiffness compared to White adults [50], possibly reflecting the effects of systemic racism in healthcare that leads to their underlying higher risk of CVD, even prior to developing measurable increases in classical CVD risk factors. In a longitudinal cohort study, the impact of certain risk factors on PWV, including lipids and glucose, was greater among Black women [51]. In a clinical trial testing the impact of an exercise intervention on blood pressure and PWV in Black and White participants, blood pressure only improved in the White participants; but, with the intervention, PWV improved similarly in both groups [52]. Therefore, our finding that higher levels of serum K were associated with reduced arterial stiffness in the JHS population deserves further study to determine if interventions designed to raise serum K can help to reduce racial disparities in arterial stiffness and longer-term CVD risk.

Limitations of this JHS analysis include the lack of follow-up serum K measurements and the length of time between K measurements and CFPWV measurements. Thus, we were not able to capture changes in serum K or dietary K intake that may have occurred in this timeframe due to changes in diet, medication use, or other medical conditions; nor can we assess if changes in K measurements changed the association between K measures and arterial stiffness. Our use of the food frequency questionnaire as the source of dietary K is a limitation in that dietary measures with food recall instruments are subject to self-report bias [53]; this prevents a robust conclusion regarding potential associations between dietary K and arterial stiffness and CVD risk. As with all epidemiologic analyses of associations, there are potential confounders that are not accounted for in our analyses. These may include other dietary contributors to aortic stiffness, such as dietary and serum sodium and calcium, as well as measures such as renal function, and genetic factors. Additionally, while CFPWV is considered a gold standard measure of aortic stiffness, there are limitations of this measure. The external measurement of transit distance of the pulse wave only approximates what occurs within the aorta itself; and properties of the arteries included in the measurement, including the aorta, iliac, and femoral arteries, can change differently due to age and other factors over time [31]. However, while imperfect, the value of CFPWV as a predictor of CVD risk has been demonstrated by in many different cohorts [16]. The events comprising our composite CVD outcome were adjudicated at different time periods, particularly heart failure vs all others, and some events prior to 2005 could have been missed or not recorded, leading to potential bias in estimated effects. However, we found similar null findings when assessing CVD events separately. More work is needed to understand the association between potassium and incident CVD events. Our data were also limited to individuals living in a single metropolitan area in the US, so our results are not generalizable beyond this population. Lastly, we performed multiple subgroup analyses *post hoc* after the main findings were known, therefore these results would require replication in a separate cohort to be able to draw firm conclusions. Strengths of this study include the large size of the cohort and the measurement of many covariates that impact CVD risk. Additionally, this cohort has the measurement of the gold standard for arterial stiffness using CFPWV in a relatively large sample size.

Based on prior studies and the results of these analyses of the JHS cohort, there are suggestive data to support the hypothesis that identification and correction of moderate K deficiency could help to reduce arterial stiffness and potentially mitigate CVD risk, particularly in Black Americans who are disproportionately impacted by CVD. Clinical trials are warranted to determine the impact of K supplementation, either dietary or pharmacologic, both of which are simple and inexpensive interventions, on these important outcomes.

## JHS disclaimer

The views expressed in this manuscript are those of the authors and do not necessarily represent the views of the National Heart, Lung, and Blood Institute; the National Institutes of Health; or the U.S. Department of Health and Human Services.

## Data sharing

Jackson Heart Study data may be requested from the Biologic Specimen and Data Repository Information Coordinating Center (BioLINCC) repository and the database of Genotypes and Phenotypes (dbGaP). Additionally, investigators with a manuscript proposal or ancillary study proposal that has been approved by study committees may request data directly from the Jackson Heart Study Coordinating Center.

BioLINCC: https://biolincc.nhlbi.nih.gov/studies/jhs/ dbGaP: https://www.ncbi.nlm.nih.gov/gap/

Jackson Heart Study Coordinating Center: https://www.jacksonheartstudy.org/Research/Study-Data/Data-Access

## Authors agreement statements

### Sources of funding

The Jackson Heart Study (JHS) is supported and conducted in collaboration with Jackson State University (HHSN268201800013I), Tougaloo College (HHSN268201800014I), the Mississippi State Department of Health (HHSN268201800015I) and the University of Mississippi Medical Center (HHSN268201800010I, HHSN268201800011I and HHSN268201800012I) contracts from the National Heart, Lung, and Blood Institute (NHLBI) and the National Institute for Minority Health and Health Disparities (NIMHD).

Dr. Davenport was partially supported by: Duke Clinical and Translational Science Institute UL1TR002553.

None of the funders contributed to this manuscript.

## Authorship requirements

all authors fulfill the role as an author

## Disclaimers

None

## CRediT authorship contribution statement

**Ranee Chatterjee:** Writing – review & editing, Writing – original draft, Investigation, Formal analysis, Conceptualization. **Clemontina A Davenport:** Writing – review & editing, Writing – original draft, Formal analysis, Data curation. **Ervin R. Fox:** Writing – review & editing, Writing – original draft, Conceptualization. **Ramachandran S. Vasan:** Writing – review & editing, Writing – original draft, Conceptualization. **Gary F Mitchell:** Writing – review & editing, Writing – original draft, Conceptualization.

## Declaration of competing interest

The authors declare the following financial interests/personal relationships which may be considered as potential competing interests:

Gary F Mitchell reports a relationship with Cardiovascular Engineering, Inc that includes: employment. Gary F Mitchell has patent pending to Gary F Mitchell. If there are other authors, they declare that they have no known competing financial interests or personal relationships that could have appeared to influence the work reported in this paper.

## Appendix Supplementary materials

Image, application 1
