# Supplementary material for: Associations between potassium, arterial stiffness, and risk of cardiovascular disease in the Jackson Heart Study: Potassium, arterial stiffness, and CVD risk
Source: Am J Prev Cardiol. 2025 Mar 8;22:100955. doi: 10.1016/j.ajpc.2025.100955 (PMC11952867; doi:10.1016/j.ajpc.2025.100955)
Supplement: Supplementary file 1 [file mmc1.docx]

**Supplementary Figure 1:** CONSORT diagram for Associations between potassium, arterial stiffness, and risk of cardiovascular disease in the Jackson Heart Study

JHS Participants

N=5306

Analytic Cohort

N=4035

- 1211 history of CVD
- 60 Missing serum potassium

Visit 1 peripheral

pulse pressure

N=3972

Tonometry peripheral

pulse pressure

N=2368

Carotid-femoral pulse wave velocity

N=1833

Incident CVD

N=3932

- 6 no visit 1 peripheral pulse pressure
- 8 no ECG heart rate
- 36 no statin use
- 9 no smoking status
- 2 no diabetes status
- 2 no BMI
- 1599 no tonometry peripheral pulse pressure
- 42 no tonometry heart rate
- 22 no statin use
- 3 no smoking status
- 1 no BMI
- 2173 no carotid-femoral pulse wave velocity
- 11 no tonometry heart rate
- 17 no statin use
- 1 no BMI
- 8 no ECG heart rate
- 36 no statin use
- 9 no smoking status
- 2 no diabetes status
- 2 no BMI
- 6 no visit 1 mean arterial pressure

Visit 1 peripheral

pulse pressure

N=3636

Carotid-femoral pulse wave velocity

N=1697

Incident CVD

N=3636

- 336 no dietary potassium
- 136 no dietary potassium
- 336 no dietary potassium

Serum Potassium Models

Dietary Potassium Models
